# Increasing extracellular matrix collagen level and MMP activity induces cyst development in polycystic kidney disease

**DOI:** 10.1186/1471-2369-13-109

**Published:** 2012-09-11

**Authors:** Bin Liu, Chenghai Li, Zijuan Liu, Zonghan Dai, Yunxia Tao

**Affiliations:** 1 Internal Medicine, Texas Tech University Health Sciences Center, 79106 Amarillo, TX, USA; 2 Institute of Life Science and Biology, Hunan University, Hunan, People’s Republic of China; 3 Henan Provincial Centers for Disease Control and Prevention, Zhengzhou, People’s Republic of China

**Keywords:** Collagen I, 3 dimensional (3D) collagen gel culture, Doxycycline, Matrix metalloproteinase, PCK rats, Polycystic kidney disease

## Abstract

**Background:**

Polycystic Kidney Disease (PKD) kidneys exhibit increased extracellular matrix (ECM) collagen expression and metalloproteinases (MMPs) activity. We investigated the role of these increases on cystic disease progression in PKD kidneys.

**Methods:**

We examined the role of type I collagen (collagen I) and membrane bound type 1 MMP (MT1-MMP) on cyst development using both *in vitro* 3 dimensional (3D) collagen gel culture and *in vivo* PCK rat model of PKD.

**Results:**

We found that collagen concentration is critical in controlling the morphogenesis of MDCK cells cultured in 3D gels. MDCK cells did not form 3D structures at collagen I concentrations lower than 1 mg/ml but began forming tubules when the concentration reaches 1 mg/ml. Significantly, these cells began to form cyst when collagen I concentration reached to 1.2 mg/ml, and the ratios of cyst to tubule structures increased as the collagen I concentration increased. These cells exclusively formed cyst structures at a collagen I concentration of 1.8 mg/ml or higher. Overexpression of MT1-MMP in MDCK cells significantly induced cyst growth in 3D collagen gel culture. Conversely, inhibition of MMPs activity with doxycycline, a FDA approved pan-MMPs inhibitor, dramatically slowed cyst growth. More importantly, the treatment of PCK rats with doxycycline significantly decreased renal tubule cell proliferation and markedly inhibited the cystic disease progression.

**Conclusions:**

Our data suggest that increased collagen expression and MMP activity in PKD kidneys may induce cyst formation and expansion. Our findings also suggest that MMPs may serve as a therapeutic target for the treatment of human PKD.

## Background

Polycystic kidney disease (PKD) is the most common genetic disorder of the kidneys and is characterized by the progressive formation and enlargement of fluid-filled cysts. These cysts compress the normal adjacent parenchyma, ultimately leading to end-stage renal disease that requires dialysis and renal transplantation [1,2]. The two major inherited forms of PKD are Autosomal Dominant PKD (ADPKD) and Autosomal Recessive PKD (ARPKD). ADPKD is caused by a mutation in either the *PKD1* or *PKD2* genes and is manifested in adulthood with an incidence of 1:1000. ARPKD is caused by mutation in *PKHD1* gene and is responsible for childhood cystic disease with an incidence of 1:20,000 [3,4]. Although remarkable progress has been made in understanding the function of the PKD genes, the molecular and cellular mechanisms that lead to cyst formation and enlargement in PKD kidneys are still not fully understood.

Growing evidence suggests that abnormal extracellular matrix (ECM) remodeling may represent a common pathway for cyst formation and development in ADPKD and ARPKD kidneys. Normal ECM remodeling is tightly regulated by dynamic synthesis and degradation of its components. This tight regulation is essential for proper morphogenesis during embryo development and for maintaining tissue and organ structure homeostasis in the adult [5,6]. During kidney development, ECM undergoes active remodeling and the levels of both, collagens, the main components of ECM, and MMPs, the main enzymes that degrade ECM components, are dynamically regulated. In particular, MMP2, MMP9, and MT1-MMP have been shown to play an important role in regulating local ECM remodeling during ureteric bud (UB) branching morphogenesis in kidney development [7,8].

Studies have shown that renal interstitial fibrosis, which is caused by excessive accumulation and deposition of collagens, is a characteristic feature of both ADPKD and ARPKD. There is a strong correlation between the progression of interstitial fibrosis and the progression of cystic disease [9,10]. In addition, overexpression of ECM collagens has been observed in cultured PKD cells as well as in PKD kidneys of human patients and animal models [11-14]. Finally, gene expression profiling of cystic kidney tissues from both human PKD patients and animal PKD models has shown that a large portion of upregulated genes encode for ECM proteins [15,16].

MMPs are key enzymes in ECM remodeling and their overexpression is another characteristic feature of PKD kidneys. Among the MMP family proteins, MMP2, MMP9 and MT1-MMP are the main collagen degradation enzymes. It has been shown that the expressions of these specific MMPs are increased in both PKD patients and PKD animal models [17-19]. MT1-MMP has been shown to play a unique role in modifying pericellular collagen microenvironment and in regulating pro-MMP2 and pro-MMP9 activation. In addition to its collagen degradation activity, MT1-MMP has also been shown to be a potent mitogen for cell proliferation [20-22]. Increased cell proliferation and dynamic remodeling of the microenvironment is a prerequisite for cyst enlargement, suggesting that MT1-MMP may be a major contributor in cyst growth.

In the present study, we investigated the role of collagen I and MT1-MMP in cyst formation and growth using both *in vitro* 3D collagen I culture system and *in vivo* PCK rat model of PKD. First, our studies reveal that the initiation of cyst structures requires higher collagen levels than that required for the formation of tubule structures. Secondly, we find that MT1-MMP stimulates cell proliferation and cyst enlargement. Finally, we show that the treatment of PCK rats with doxycycline, an FDA approved pan-MMPs inhibitor, significantly inhibited cystic disease progression in polycystic kidneys. Taken together, our results suggest that increased collagen expression and MMP activity in PKD kidneys may play a crucial role in renal cyst formation and enlargement.

## Methods

### PCK rat model

3 to 4 weeks old male PCK rats were purchased from Charles River (Wilmington, MA). The protocol for animal studies has been approved by the Texas Tech University Health Sciences Center Animal Care and Use Committee. Rats had free access to tap water and standard rat chow. Male PCK rats were treated at 4 weeks of age daily with either doxycycline (Sigma, St. Louis, MO) at 30 mg/kg or vehicle (PBS) subcutaneously for 5 weeks.

### Immunohistochemical staining

OCT-embedded kidney sections (5 μm) were used for the immunohistochemical studies. For antigen retrieval, the sections were first immersed in heated (95°C to 100°C) 0.1 M citrate buffer, pH 6.0 for 10 min and then allowed to cool to room temperature. The sections were then incubated with the antibodies against collagen I (Santa Cruz Biotechnology, Santa Cruz, CA), MMP2 (Millipore, Temecula, CA), MMP9 (Santa Cruz Biotechnology, Santa Cruz, CA) or MT1-MMP (Thermo Fisher Scientific, Fremont, CA), respectively. After extensive wash, the sections were incubated with horseradish peroxidase (HRP)-conjugated polymer (DAKO EnVision System, Carpinteria, CA) for 1 h, and the antigen sites were visualized by incubating with the substrate - DAB.

### Cell culture and transfections

MDCK cells were obtained from American Type Culture Collection (Manassas, VA) and grown in Dulbecco’s modified Eagle’s medium (DMEM)/Ham’s F12 (50:50) medium supplemented with 10% fetal bovine serum (FBS), 100 U/ml penicillin, and 100 U/ml streptomycin (Mediatech, Manassas, VA). pMSCVGFP-MT1-MMP expression vector is a gift from Dr. Zonghan Dai (Texas Tech University HSC). Transfections of MDCK cells with pMSCVGFP-MT1-MMP or pMSCVGFP control vector were performed using Lipofectamine 2000 (Invitrogen, Carlsbad, CA), as described by manufacturer’s procedure and the stably transfected clones were selected by 5 μg/ml puromycin.

### RNA isolation and RT-PCR

Total RNA was isolated using RNeasy Mini Kit (Qiagen, Valencia, CA), as described in the manufacturer’s instructions. cDNA was synthesized using SuperScript III First-strand Synthesis System (Invitrogen, Carlsbad, CA). The RT-PCR was performed using the following primers: MT1-MMP forward primer: 5′-GGATACCCAATGCCCATTGGCCA-3′, reverse primer: 5′-CCATTGGGCATCCAGAAGAGAGC-3′; 28S RNA forward primer: 5′-TTGAAAATCCGGGGGAGAG-3′, and reverse primer: 5′-ACATTGTTCCACATGCCAG-3′.

### 3D collagen gel culture and treatment

Collagen I was obtained from BD Biosciences (San Jose, CA). Collagen I concentration was determined using Modified Lowry Protein Assay Kit (Thermo Scientific, Pittsburgh, PA). The collagen I solutions with desired concentration in DMEM containing 100 U/ml penicillin, 100 U/ml streptomycin, 10 mM Hepes (pH 7.2) and 1.2 mg/ml NaHCO_3_ were prepared on ice, and then mixed with cells to make a cell-collagen suspension at a density of 1 × 10^4^ cells/ml. Aliquots of 0.4 ml of the cell-collagen suspension were plated in a 24-well plate and incubated for 30 min in a 37°C, 5% CO_2_ incubator to allow collagen solidified. Complete culture medium was then added on the top of collagen gel and cultured for 9 days. To test the effect of MMPs on cyst growth, the cultures were treated with doxycycline at a concentration of 12.5ug/ml. To view the histological structure of the cysts and tubules, the 3D cultures were embedded in OCT at the end of experiment.

### Western blotting analysis

Lysates from 2D cultures were prepared as described previously [23]. To prepare lysates from 3D cultures, the collagen gels were washed with PBS for 3 times and then solubilized in RIPA buffer (1% Triton X-100, 1% deoxycholate, 0.1% sodium dodecyl sulfate, 20 mM Tris–HCl, pH 7.4, 0.16 M NaCl, 1 mM EGTA, 1 mM EDTA, and 15 mM sodium fluoride) containing protease inhibitor cocktails (Sigma, St. Louis, MO). The mixtures were kept on ice for 1 h with periodically vortexing and then, centrifuged at 6000 rpm for 10 min at 4°C. The supernatants were collected as cell lysates. Because the 3D culture lysates prepared by this method may contain collagen I, we first normalized these lysates for β-actin contents by Western blotting analysis. The lysates containing equal amount of β-actin were then separated on a 10% SDS-PAGE gel and transferred to a PVDF membrane (Bio-Rad, Hercules, CA). The membranes were probed with the antibodies against GFP (Chemicon, Temecula, CA) and PCNA (Santa Cruz Biotechnology), respectively. To confirm equal loading, the membranes were also probed with anti-β-actin antibody (Sigma, St. Louis, MO). After incubated with HRP-conjugated secondary antibodies, the immunoreactive proteins were visualized by ECL detection system (Amersham Biosciences Piscataway, NJ).

### Cyst and tubule structure measurement and statistical analysis

Images of cysts and tubules in 3D collagen gels were captured and analyzed using an inverted microscope (Nikon Eclipse TE 2000-U). This was performed by a reviewer who was blinded to the identity of the treatment modality. At least ten random areas were captured for each treatment condition. The diameters of the cysts were measured using NIS-Elements BR 2.30 software and the mean and standard deviation were determined using Excel software. Comparison of the means was performed with the Student’s *t*-test. The P value < 0.05 was considered statistically significant. Values are expressed as mean ± STDEV.

### Cyst volume density and kidney function

Hematoxylin-eosin-stained kidney sections cut longitudinally through the midline were used to determine the cyst volume density by point counting stereology [24]. The entire kidney section was counted to guard against field selection variation. Serum creatinine levels were measured using Creatinine Assay kit (BioVison, Mountain View, CA). Blood urea nitrogenBlood urea nitrogen levels were measured using MaxDiscovery^™^ Blood Urea Nitrogen Enzymatic Assay Kit (B100 Scientific Corporation, Austin, TX).

## Results

### Expression of Collagen I, MMP2, MMP9 and MT1-MMP are increased in PKD kidneys of PCK rats

**Figure 1:**
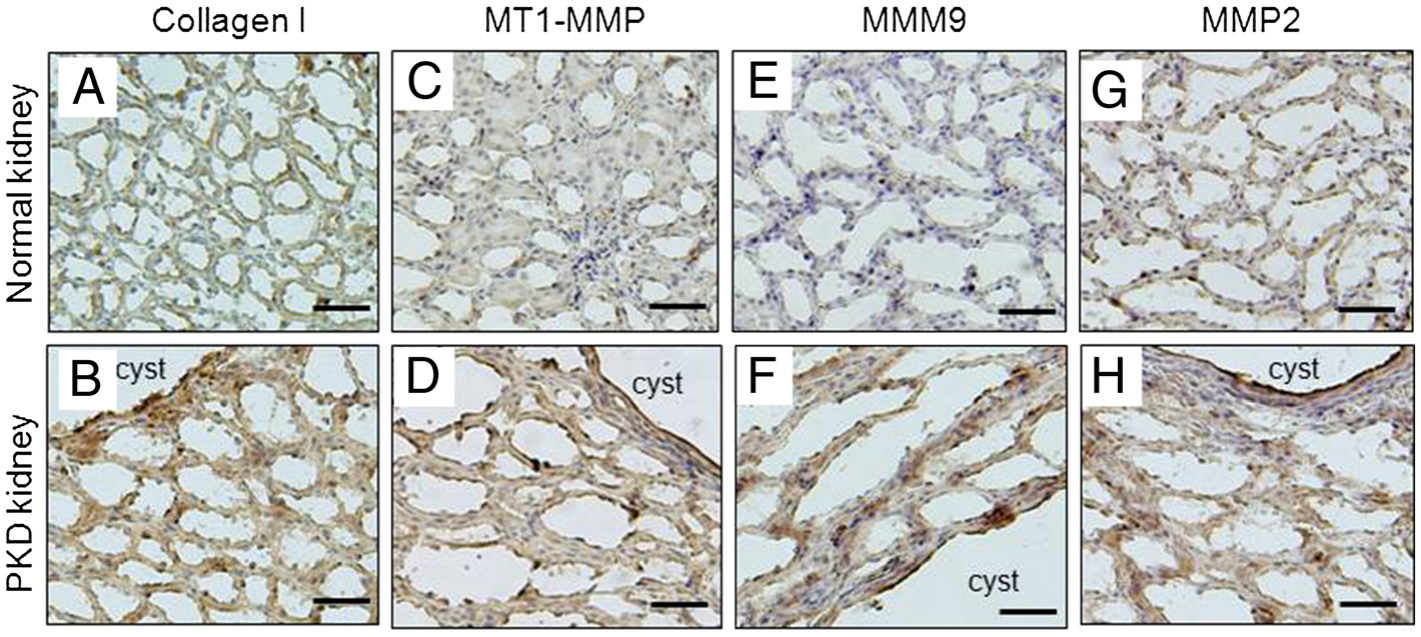
**The levels of collagen I, MMP9, MMP2 and MT1-MMP are increased in PKD kidneys of PCK rats.** Immunohistochemical staining of collagen I, MT1-MMP, MMP9 and MMP2 (brown) of 9 weeks old normal rat kidney (top panel, **A**, **C**, **E** and **G**) and PKD kidneys (bottom panel, **B**, **D**, **F**, and **H**) of PCK rats (Scale bars, 50 μm).

Immunohistochemical staining for collagen I, MMP2, MMP9, and MT1-MMP was performed on PKD kidneys harvested from 9 weeks old male PCK rats. Age-matched male Sprague–Dawley rats were used for control. Figure 1 A and B shows that collagen I expression is dramatically increased in PKD kidneys compared to control. The high collagen I expression was observed in pericystic areas as well as non-cystic, interstitial areas of the PKD kidneys. Similarly, the expression of MT1-MMP, MMP9 and MMP2 are all significantly increased relative to the low levels expressed in control kidneys (Figures 1C to 1H). Expression of these MMPs is increased in the cyst epithelial cell areas as well as in the interstitial of non-cystic tubule cells.

### Collagen concentration determines the formation of cyst or tubular structure in 3D culture

**Figure 2:**
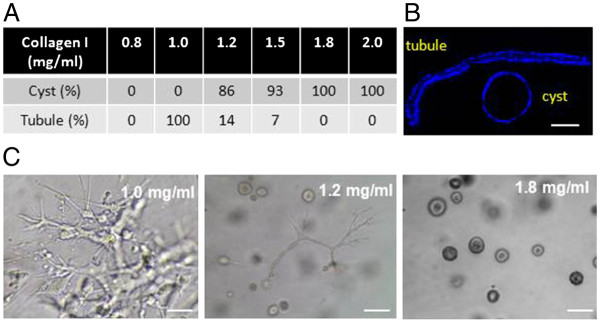
**Effect of collagen I concentrations on the formation of cyst or tubule structures in 3D collagen I gel cultures. A **. percentage of cyst and tubule structures formed in 3D collagen gels with a collagen I concentration of 0.8, 1.0, 1.2, 1.5, 1.8 and 2.0 mg/ml, respectively. **B**. a representative tubule and cyst section stained with DAPI to show the single cell layer of tubule and cyst structures (Scale bar, 50 μm). **C**. a representative picture of cyst and tubule structures formed in 1.0, 1.2, and 1.8 mg/ml collagen I gels (Scale bars, 200 μm).

MDCK cells were cultured in 3D collagen gels at varying collagen I concentrations to examine the effects of collagen I concentrations on cyst formation and growth. MDCK cells cultured in collagen I concentrations below 1.0 mg/ml do not form 3D structures; 3D structures become apparent at concentrations exceeding 1.0 mg/ml. MDCK cells form tubules in a 1.0 mg/ml 3D collagen I gel. A mixture of cyst and tubule structures is formed when cells are cultured in 1.2 mg/ml 3D collagen I gel. It is important to note that the cyst to tubule ratio increases as collagen I concentrations increase. Remarkably, only cyst structures are formed when collagen I concentration reached to 1.8 mg/ml (collagen threshold for cyst formation) (Figure 2A). These cyst and tubule structures were confirmed by microscopy analysis of DAPI-stained OCT sections (Figure 2B). Figure 2C is a representative picture of cyst and tubule structures formed at collagen I concentrations of 1.0, 1.2, and 1.8 mg/ml.

### MT1-MMP stimulates cell proliferation and cyst expansion in 3D collagen gel cultures

**Figure 3:**
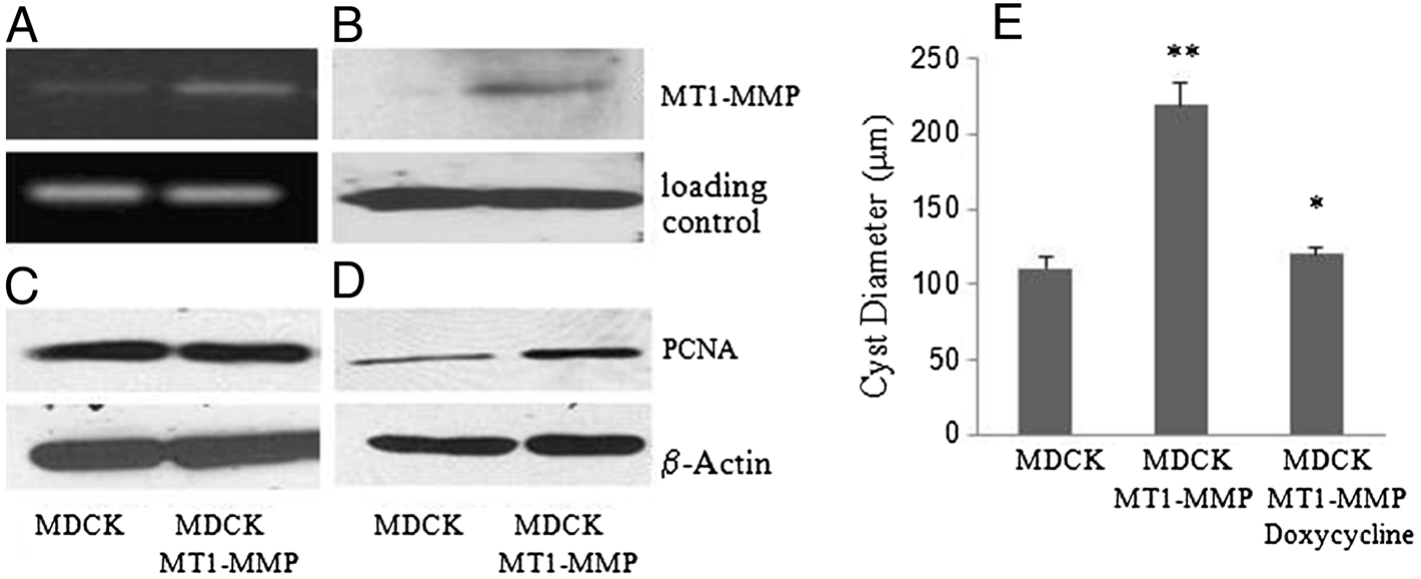
**MT1-MMP induces cell proliferation and cyst growth in 3D collagen I gel culture. A ** and **B**: RT-PCR (**A**) and western blot (**B**) analyses of the MT1-MMP mRNA and protein expression in parental and MT1-MMP overexpressed MDCK cells. **C** and **D**: Western blot analysis of the PCNA expression in parental and MT1-MMP overexpressed MDCK cells that were cultured under 2D (**C**) or 3D (**D**) conditions. **E**. The diameters of the cysts that were formed from parental MDCK cells or MT1-MMP overexpressing MDCK cells treated with or without Doxycycline for 9 days in 2.5 mg/ml collagen I gels. **p < 0.005 versus MDCK cells. *p < 0.005 versus MDCK MT1-MMP cells.

To investigate the role of increased MMPs in PKD kidneys in cyst enlargement, we transfected MDCK cells with a MT1-MMP expression vector (MDCK-MT1-MMP cell). The overexpression of MT1-MMP in MDCK cells was confirmed at both mRNA (Figure 3A) and protein (Figure 3B) levels.

Western blot analysis of proliferating cell nuclear antigen (PCNA), a molecular marker for proliferating cells, shows that the overexpression of MT1-MMP in MDCK cells did not increase PCNA levels under a 2D culture condition (Figure 3C). However, expression of PCNA is significantly increased in 3D collagen I gel cultures (Figure 3D). Moreover, the overexpression of MT1-MMP in MDCK cells resulted in a shift of the collagen concentration threshold for cyst formation from 1.8 mg/ml to 2.5 mg/ml. To study the impact of increased MT1-MMP expression on cyst enlargement, we compared the growth of the cysts formed by parental MDCK cells to that of the cysts formed by MDCK-MT1-MMP cells in 3D gels with a collagen concentration of 2.5 mg/ml, at which both cell lines form cysts predominantly. We found that the overexpression of MT1-MMP significantly increased cyst growth. Furthermore, the cyst growth was attenuated by the addition of doxycycline (Figure 3E).

### Inhibition of MMPs slows cystic disease progression and kidney enlargement

**Figure 4:**
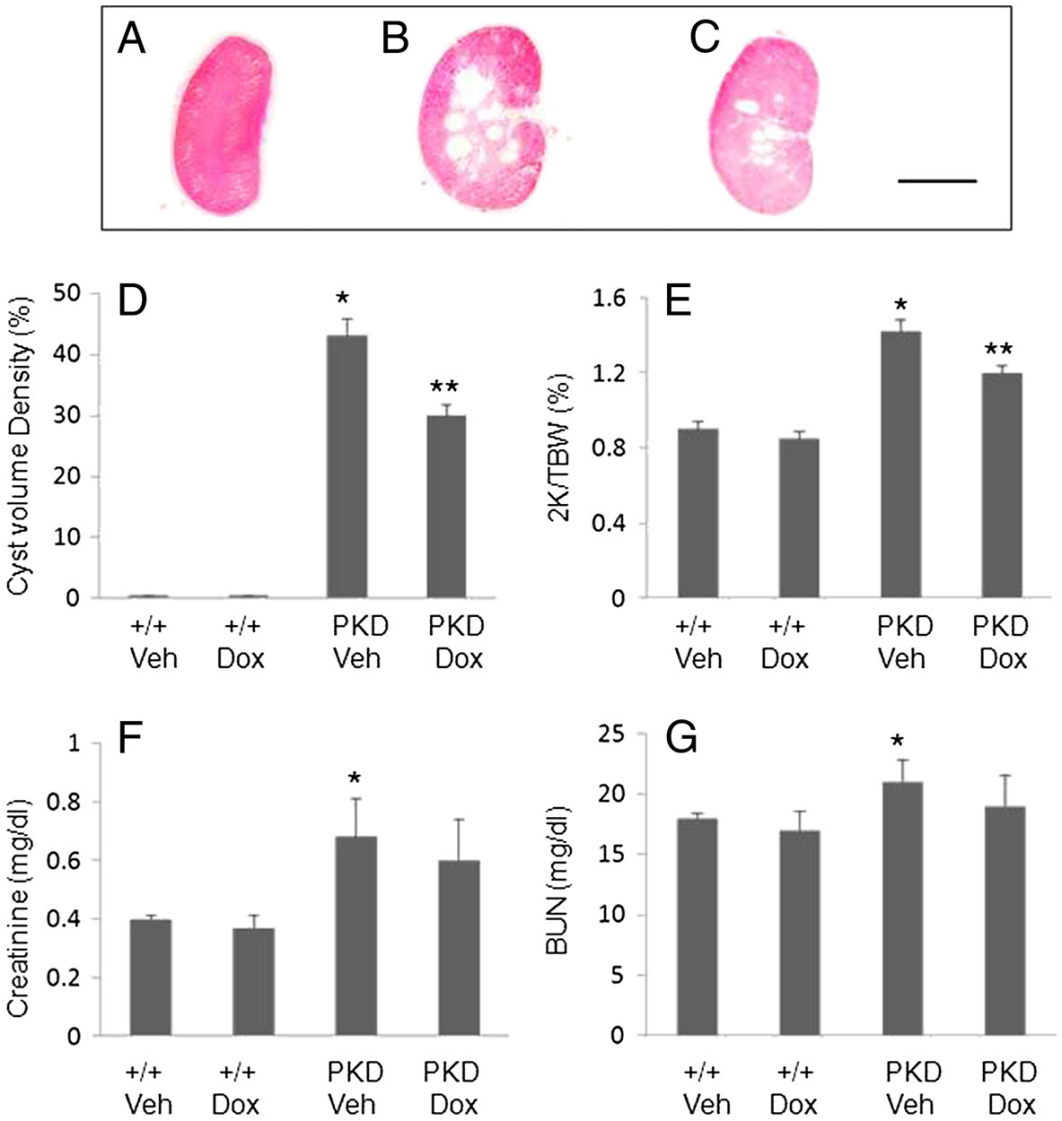
**Effects of doxycycline (Dox) on the development of PKD in PCK rats. A ** to **C**. Representative kidney sections from the vehicle-treated control (**A**) and PCK (**B**) rats as well as the doxycycline-treated PCK rat (**C**) (Scale bar, 1.5 cm). **D**. Doxycycline reduced the cyst volume of the PKD kidneys. The cyst volume density (CVD) of the kidneys from the vehicle-treated control (+/+ Veh) and PCK (PKD Veh) rats, and from the doxycycline-treated control (+/+ Dox) and PCK (PKD Dox) rats was measured. The doxycycline treatment did not affect CVD in +/+rats but decreased it in the PCK rats. *P < 0.001 versus +/+ vehicle, **p < 0.005 versus PKD vehicle. **E**. Doxycycline reduced the kidney enlargement in PCK rats. The two-kidney/total body weight ratio (2 K/TBW) was examined in the vehicle-treated control (+/+ Veh) and PCK (PKD Veh) rats and were compared to that of doxycycline-treated control (+/+ Dox) and PCK (PKD Dox) rats, respectively. The doxycycline treatment did not affect 2 K/TBW in +/+rats but decreased it in the PCK rats. *p < 0.001 versus +/+vehicle, **p < 0.005 versus PKD vehicle. **F**. Doxycycline treatment did not significantly affect creatinine level. The creatinine levels were measured in the vehicle-treated control (+/+Veh) and PCK (PKD Veh) rats and were compared to those in the doxycycline-treated control (+/+ Dox) and PCK (PDK Dox) rats. *p < 0.001 versus +/+ vehicle. **G**. BUN was unaffected by doxycycline in +/+rats. The PCK rats at 9 weeks of age have a slight increase in BUN (*p < 0.005 versus +/+ vehicle); however, the doxycycline treatment did not significantly affect BUN level.

4 week-old male PCK rats were treated daily with 30 mg/kg doxycycline or vehicle for 5 weeks. The body weight was 373 ± 7 g in vehicle-treated rats (n = 5) and 331 ± 11 g in doxycycline-treated rats (n = 5). Despite the 11% decrease in body mass, all of the rats seemed healthy during the treatment. The cyst volume density (CVD) in vehicle-treated PKD kidneys reached to 43% and doxycycline reduced CVD to 30% (Figure 4D). Figure 4A to C shows representative kidney sections through midline of vehicle-treated normal rat kidney, vehicle-treated PCK rat kidney, and doxycycline-treated PCK rat kidney. The two-kidney/total body weight ratio (2 K/TBW) was determined to correct for the lower body mass caused by the doxycycline. The 2 K/TBW ratio was 0.90 ± 0.04 (n = 8) in vehicle-treated +/+, 0.85 ± 0.04 in doxycycline-treated +/+ rats (n = 5), 1.42 ± 0.06 in vehicle-treated PCK rat kidney and 1.20 ± 0.04 in doxycycline-treated PCK rat (Figure 4E). PCK rats had a 60% increase in kidney size compared with +/+ control. Doxycycline reduced the kidney enlargement by 56%.

The serum creatinine levels were 0.4 ± 0.01 and 0.4 ± 0.05 in vehicle- and doxycycline-treated +/+control rats, 0.68 ± 0.13 and 0.59 ± 0.14 in vehicle- and doxycycline-treated PCK rats, respectively (Figure 4F). BUN levels were 18 ± 0.4 and 17.3 ± 1.6 in vehicle- and doxycycline-treated +/+control rats, 21 ± 1.8 and 19 ± 2.6 in vehicle- and doxycycline-treated PCK rats, respectively (Figure 4G). These results show that PCK rats at 9 weeks of age had a slightly increased creatinine and BUN levels compared to the age-matched control animals (P < 0.001 and P < 0.005, respectively), but the treatment with doxycycline did not significantly affect creatinine and BUN levels.

**Figure 5:**
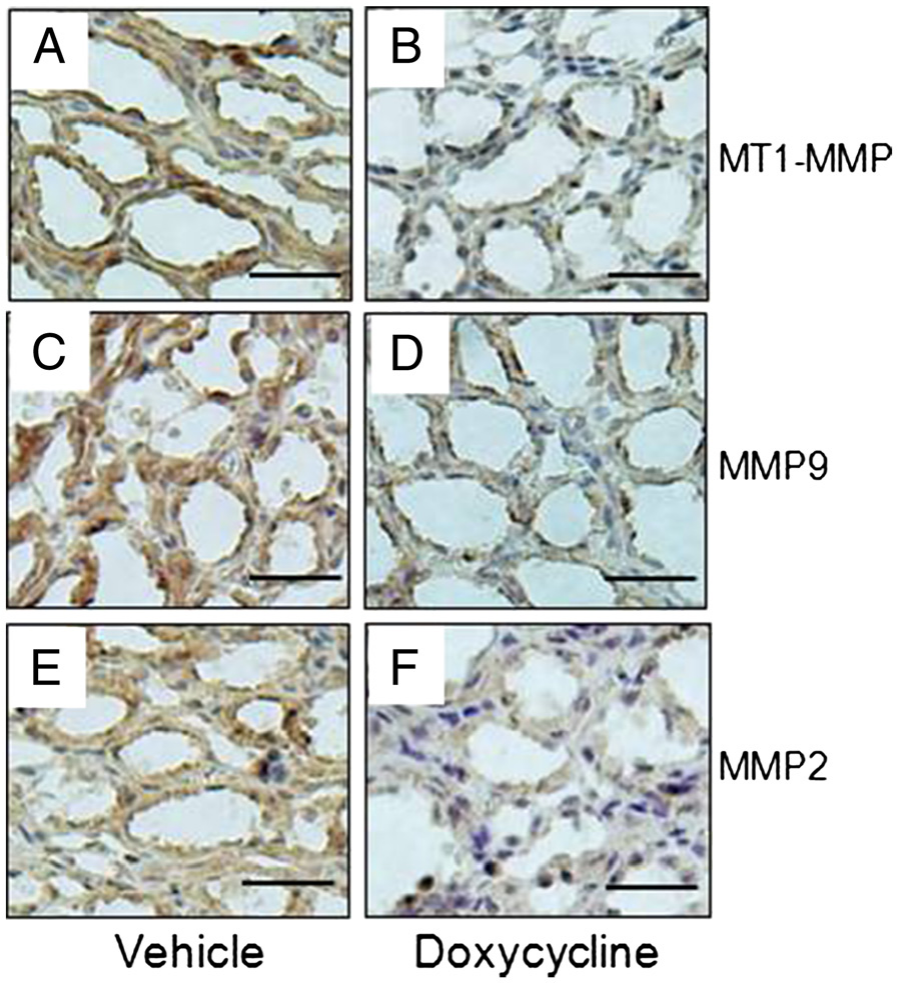
**Doxycycline inhibits MT1-MMP, MMP9 and MMP2 expression in polycystic kidneys of PCK rats.** Immunohistochemical staining (brown color) of vehicle-treated (**A**, **C**, **E**) and doxycycline-treated kidneys (**B**, **D**, **F**) of PCK rats with antibodies against MT1-MMP, MMP9, and MMP2, respectively (Scale bars: 50 μm).

**Figure 6:**
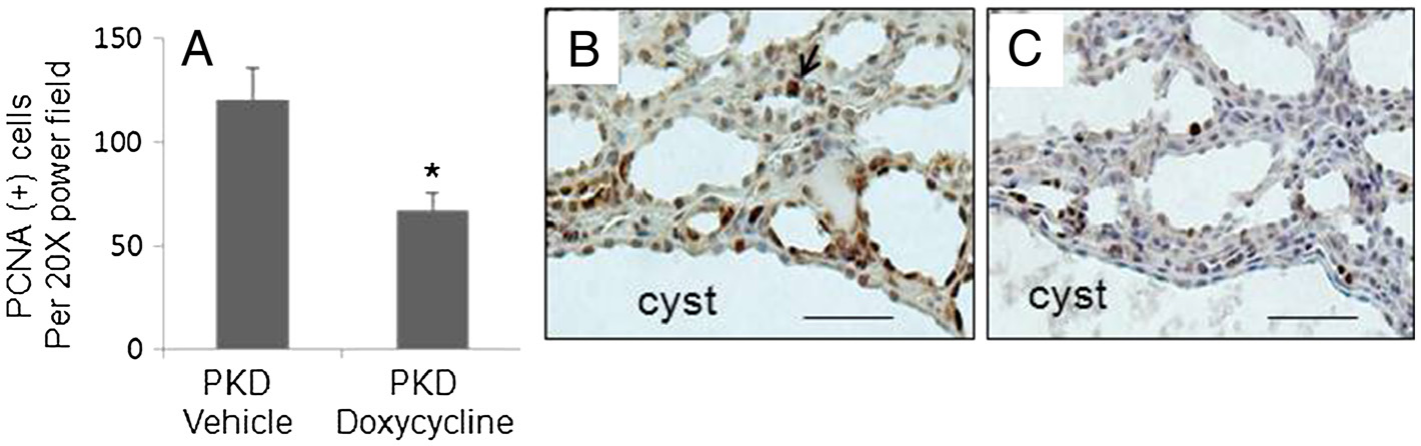
**Doxycycline decreases the number of PCNA positive tubule/cyst cells in PKD kidneys of PCK rats. A **. The number of PCNA-positive cells per 20X power field in vehicle- and doxycycline-treated PKD kidneys. *p < 0.005 versus PKD vehicle. **B** and **C**. The representative pictures of PCNA nuclear staining (brown color) in vehicle- (**B**) and doxycycline-treated (**C**) PKD kidneys (Scale bars: 50 μm).

Immunohistochemical staining shows that the expressions of MT1-MMP, MMP9 and MMP2 were significantly lower in doxycycline-treated PKD kidney compared to vehicle-treated controls (Figure 5). The number of PCNA-positive kidney tubule/cyst cells per 20 X power field was 121 ± 15 in vehicle-treated PCK kidneys and 67 ± 9 in doxycycline-treated PCK rat (Figure 6). Representative pictures are shown in Figure 6 B and C.

## Discussion

Studies in human PKD patients and several PKD animal models including Han: SPRD rats and pcy mice showed that abnormal ECM remodeling, such as increased collagen I level and MT1-MMP expression, are the characteristic feature of PKD kidneys [15,16]. However, the direct link of increased ECM collagen and MT1-MMP on cyst formation and enlargement in PKD kidneys has not been well described.

In this study, we investigated the role of increased collagen I and the collagenase MMPs on cystic kidney disease progression using both *in vivo* PCK rats and *in vitro* 3D collagen I cyst culture systems. PCK rats are orthologous model of human ARPKD and have been successfully used for testing several potential therapeutic agents, therefore provide one of the best animal models for studies of human PKD [25,26]. A recent study has shown that there is an epithelial-to-mesenchymal transition in cyst-lining cells of PCK rats, suggesting that increased ECM production may occur in the kidneys [27]. However, the expression of ECM collagen and MMPs in PKD kidneys of PCK rats has not been previously described. We show here that the expression of collagen I, MMP2, MMP9 and MT1-MMP in PKD kidneys of 9 week old PCK rats are all significantly increased compared to the age-matched normal control rats. This finding further supports the proposition that abnormal ECM metabolism is a common feature of PKD. We found that increased collagen and MMPs expression not only occurred in cyst-lining epithelial cells, but also in the interstitial area in PKD kidneys. These observations are in agreement with the studies of pcy mice model by Okada et al, showing that both cystic epithelial cells and interstitial fibroblasts express higher levels of collagens compared to normal controls [10].

3D collagen I gel is a well-established culture system for studies of epithelial cell tubule and cyst morphogenesis. However, past studies have primarily focused on the role of signaling molecules, such as HGF and cAMP on cyst and tubule structure development [28,29]. No studies have been conducted to directly examine the effect of collagen concentrations and MMPs activities on cyst and tubule morphogenesis of cultured cells. We found that the formation of cyst structure requires a collagen concentration higher than that required for the formation of tubular structure. Given that the increased collagen expression has been detected in cystic epithelial cells and adjacent fibroblasts, our results suggest that this locally increased collagen production in PKD kidneys may play a critical role in the initiation of cyst formation.

Finally, we assessed the effects of MMPs inhibition by doxycycline on cystic disease progression in a PKD animal model, PCK rat. Doxycycline, also known as Periostat, is currently the only FDA approved MMPs inhibitor. Doxycycline is used to treat several disorders that are mediated by increased MMP activities such as cancer and periodontal disease. It has been shown to be potent in inhibiting the activities of a large number of MMPs, including MMP2, MMP9, and MT1-MMP [30]. We show that doxycycline significantly inhibited MT1-MMP, MMP9 and MMP2 expression and slowed cyst disease progression in PCK rats. We also demonstrate that doxycycline inhibits cyst growth in 3D collagen gel. Increased cell proliferation is prerequisite to cyst expansion and MT1-MMP has been shown to stimulate cell proliferation in both *in vivo* and *in vitro* studies. In this study, we showed that doxycycline inhibits MT1-MMP expression and cell proliferation in PKD kidneys. The effect of MMPs inhibition on PKD progression has been previously studied by Obermuller et al in Han:SPRD PKD rats using batimastat, a synthetic inhibitor of MMPs [31]. Their study show that the batimastat treatment significantly decreased cyst number but not the total cyst volume density in PKD kidneys. They proposed that this is likely due to that the dosage of batimastat used in their study is too low to effectively block the high amounts of MT1-MMP being expressed in large cysts and therefore, to inhibit large cyst expansion. They also indicate that the application of higher doses of batimastat may increase side effect, in particular, renal dysfunction. In our study, we show that the dosage of doxycycline used in our experiments not only efficiently inhibits overall cyst volume density in PKD kidney of PCK rats, but also avoid any apparent kidney function damage and other side effects.

## Conclusions

Our *in vivo* and *in vitro* data suggest that abnormal increase in collagen and collagenase MMPs expression may play an important role in cyst formation and enlargement in PKD kidneys. The finding that doxycycline markedly inhibits cyst growth in PCK rats suggests a potential use of this drug in therapeutic intervention of the cystic disease progression. As doxycycline has been used successfully in treating human patients with several disorders that are mediated by increased MMP activities, we believe that it may provide a promising drug candidate for the treatment of human PKD patients.

## Competing interests

The authors declare that they have no competing interests.

## Authors’ contributions

BL, CL, and ZL carried out experiments and analyzed data. ZD analyzed and interpreted data and involved writing of the manuscript. YT designed the study and wrote the paper. BL and CL contributed equally to this work. All authors had final approval of the submitted and published versions.

## Pre-publication history

The pre-publication history for this paper can be accessed here:

http://www.biomedcentral.com/1471-2369/13/109/prepub

## Author details

^1^Internal Medicine, Texas Tech University Health Sciences Center, 79106 Amarillo, TX, USA. ^2^Institute of Life Science and Biology, Hunan University, Hunan, People’s Republic of China. ^3^Henan Provincial Centers for Disease Control and Prevention, Zhengzhou, People’s Republic of China.
